# Effect of Chemical Composition on Magnetic and Electrical Properties of Ferroelectromagnetic Ceramic Composites

**DOI:** 10.3390/ma14102488

**Published:** 2021-05-11

**Authors:** Dariusz Bochenek, Przemysław Niemiec, Artur Chrobak

**Affiliations:** 1 Faculty of Science and Technology, Institute of Materials Engineering, University of Silesia in Katowice, 41-500 Chorzów, Poland; przemyslaw.niemiec@us.edu.pl; 2 Faculty of Science and Technology, Institute of Physics, University of Silesia in Katowice, 41-500 Chorzów, Poland; artur.chrobak@us.edu.pl

**Keywords:** ferroelelectromagnetics, multiferroics, multiferroic composites, piezoelectrics, ferrites

## Abstract

In this paper, ferroelectric–ferrimagnetic ceramic composites based on multicomponent PZT-type (PbZr_1−*x*_Ti*_x_*O_3_-type) material and ferrite material with different percentages in composite compositions were obtained and studied. The ferroelectric component of the composite was a perovskite ceramic material with the chemical formula Pb_0.97_Bi_0.02_(Zr_0.51_Ti_0.49_)_0.98_(Nb_2/3_Mn_1/3_)_0.02_O_3_ (P), whereas the magnetic component was nickel-zinc ferrite with the chemical formula Ni_0.5_Zn_0.5_Fe_2_O_4_ (F). The process of sintering the composite compounds was carried out by the free sintering method. Six ferroelectric-ferrimagnetic ceramic P-F composite compounds were designed and obtained with different percentages of its components, i.e., 90/10 (P90-F10), 85/15 (P85-F15), 80/20 (P80-F20), 60/40 (P60-F40), 40/60 (P40-F60), and 20/80 (P20-F80). X-ray diffraction patterns, microstructural, ferroelectric, dielectric, magnetic properties, and DC electrical conductivity of the composite materials were investigated. In this study, two techniques were used to image the microstructure of P-F composite samples: SB (detection of the signals from the secondary and backscattered electron detectors) and BSE (detection of backscattered electrons), which allowed accurate visualization of the presence and distribution of the magnetic and ferroelectric component in the volume of the composite samples. The studies have shown that at room temperature, the ceramic composite samples exhibit good magnetic and electrical properties. The best set of physical properties and performance of composite compositions have ceramic samples with a dominant phase of ferroelectric component and a small amount of the ferrite component (P90-F10). Such a composition retains the high ferroelectric properties of the ferroelectric component in the composite while also acquiring magnetic properties. These properties can be prospectively used in new types of memory and electromagnetic converters.

## 1. Introduction

Currently, in modern microelectronics, the most versatile application uses are materials with functional properties [1,2]. These include piezoelectric, multiferroic materials, and ceramic composites with ferroelectric and ferromagnetic properties [3,4,5,6,7,8]. The application possibilities depend on the coupling of magnetic and electrical sub-systems in such materials [9,10,11]. Multiferroic ceramic composites are designed with the goal of achieving the appropriate physical properties in a single material, which are represented by individual composite components.

The group of multiferroic materials includes two-phase composites (e.g., with ferroelectric and magnetic phase), as well as multiphase composites, where besides the ferroelectric and magnetic phase, an additional phase, e.g., polymeric phase is introduced. With such modification, the ceramic-polymer composite obtains additional properties, i.e., it shows both high stiffness and hardness (which characterize the ceramic material) and resistance to dynamic loads (given by the polymer component), which cannot be obtained in each of the individual components of such a composite.

One way to obtain ceramic materials with functional properties is to combine piezoelectric material (for the most part) with other materials with different properties (e.g., magnetic, magnetostrictive) [12,13]. So far, the best set of performance parameters (ferroelectric, electromechanical, pyroelectric, and piezoelectric) is shown by multicomponent materials obtained based on the PbZr_1−*x*_Ti*_x_*O_3_ (PZT) solid solution. The PZT material has a perovskite structure with the general formula ABO_3_, where the A positions are occupied by lead cations Pb, while the B positions are occupied alternately by zirconium cations Zr, and titanium cations Ti [14]. PZT-type solid solutions are characterized by a broad isomorphism, which allows multiple doping and modification of the basic PZT composition [15,16]. This is one of the most effective ways of being able to efficiently improve the functional physical properties of ceramic materials with perovskite structure [17,18].

For obtaining ferroelectric-ferrimagnetic ceramic composites, various kinds of magnetic materials are used, i.e., nickel-zinc ferrites, manganese-zinc ferrites, or manganese-chromium–zinc ferrites. Ferrites obtained on the basis of ferric oxide Fe_2_O_3_ with metal oxides have the spinel structure MgAl_2_O_4_, with the general formula MeO, where Me: Zn, Cd, Fe, Ni, Cu, Co, or Mg [19], and their magnetic properties depend on their chemical composition [20]. A distinction is made between magnetically soft and magnetically hard ferrites [21], which have found numerous applications in, among others, radiotechnics, high frequency technology, ultrasound technology, and as cores for coils, chokes, transformers, magnetic antennas, etc. [22,23].

In this work, six compositions of ferroelectric-ferrimagnetic ceramic composites containing two main phases were obtained, i.e., ferroelectric phase (multicomponent PZT-type material) and magnetic phase (nickel-zinc ferrite). Individual components of the composite were selected on the basis of their high dielectric and piezoelectric properties of the component (P) and the strong magnetic properties of the nickel-zinc ferrite (F), as well as its relatively low electrical conductivity. The ceramic composite, which is a combination of two materials with different properties, makes it possible to miniaturization of a microelectronic devices, e.g., for use in new types of memory or electromagnetic converters. A comprehensive study of the basic properties of the multiferroic composites, including X-ray diffractions (XRD), scanning electron microscopy (SEM) using the backscattered electron detector (BSE), energy dispersive spectrometry (EDS), electron probe microanalysis (EPMA), as well DC electrical conductivity, dielectric, ferroelectric, and magnetic properties, was performed. We present the way in which the individual components of the composite material affect their microstructure as well as the magnetic and electrical properties.

## 2. Experiment

Ferroelectric–ferromagnetic ceramic composites were prepared by combining doped PZT-type solid solution with the chemical formula Pb_0.97_Bi_0.02_(Zr_0.51_Ti_0.49_)_0.98_(Nb_2/3_Mn_1/3_)_0.02_O_3_ (P) and ferrite powder with the chemical formula Ni_0.5_Zn_0.5_Fe_2_O_4_ (F). The technological process consisted of three main stages.

In the first stage, the ferroelectric multicomponent PZT-type material was carried out. Particular input components of the compound, i.e., oxides: PbO (99.9%, POCH, Gliwice, Poland), ZrO_2_ (99.5%, Aldrich, St. Louis, MO, USA), TiO_2_ (99.99%, Merck, Darmstadt, Germany), Bi_2_O_3_ (99.9%, Aldrich, St. Louis, MO, USA), Nb_2_O_5_ (99.9%, Sigma–Aldrich, St. Louis, MO, USA), and MnO_2_ (99%, Aldrich, St. Louis, MO, USA), were weighed in stoichiometric proportions and milled in a planetary ball mill of Fritsch Pulverisette 6 type (Idar-Oberstein, Germany), wet (in ethanol), for 15 h. The synthesis of the powders was performed by solid-phase sintering at the following conditions: 850 °C/2 h. After synthesis, the samples were ground to powder and milled again.

In the second stage, the ferrite material was carried out. The individual components of the compound, i.e., NiO (99.99%, POCH, Gliwice, Poland), Fe_2_O_3_ (99.9%, POCH, Gliwice, Poland), and ZnO (99.99%, POCH, Gliwice, Poland), were weighed in stoichiometric amounts and then mixed in a ball mill (wet in ethanol for 15 h). The synthesis of the ferrite powder Ni_0.50_Zn_0.50_Fe_2_O_4_ (F) was performed by calcination under conditions: 1100 °C/4 h [24].

The third stage of the technological process concerns the preparation of composites by connecting the materials obtained in the first and second stages. The synthesized powders of the starting components of ferroelectric-ferromagnetic composites were measured in appropriate P/F ratios: 20/80, 40/60, 60/40, 80/20, 85/25, 90/10, and then subjected to wet milling (in ethanol) in a planetary ball mill (Fritsch Pulverisette 6) for 15 h. The synthesis of composite powders was performed by calcination at 950 °C/2 h, and densification of the ceramic samples was carried out by the free sintering method (non-pressure sintering) under the conditions of 1250 °C/2 h. Ferroelectric–ferromagnetic composites were marked as follows: 90/10 (P90-F10), 85/15 (P85-F15), 80/20 (P80-F20), 60/40 (P60-F40), 40/60 (P40-F60), and 20/80 (P20-F80). After the sintering process, the surfaces of samples were ground, polished, and on their surfaces, silver electrodes were applied using the burning method.

The X-ray diffraction patterns of the ceramic composite samples were measured at room temperature (*RT*) using a X’Pert Pro diffractometer (PANalytical, Eindhoven, the Netherlands) with CuK*α* = 1.54056 Å radiations. Diffraction patterns were registered in the 2*θ* range from 10° to 60°, in steps-scan mode: 0.05° and 4s/step. Phase identification was performed based on data from the ICDD PDF-4 database (International Center for Diffraction Data Powder Diffraction Files). A scanning electron microscope JEOL JSM-7100F TTL LV (Jeol Ltd., Tokyo, Japan) was used to examine morphology microstructure of samples. The images were taken in the standard SB mode (detection of the assembly of signals from the secondary and backscattered electron detectors) as well as in the BSE technique (detection of backscattered electrons). The chemical composition tests of ceramic composite samples were performed by the standard method using an Energy Dispersive Spectrometer (EDS, JSM-7100F TTL LV Jeol Ltd., Tokyo, Japan). The EPMA (Electron Probe Microanalysis) analyses were also performed on this detector. The SEM/EDS/EPMA investigations were performed by the accelerating voltage 15–20 kV, low vacuum, and with the use of the Au sputtering technique (Smart Coater DII-29030SCTR, Jeol Ltd., Tokyo, Japan).

Dielectric properties of the composite samples (electric permittivity and dielectric loss) were measured using the LCR meter (QuadTech 1920 Precision LCR Meter, Inc., Maynard, MA, USA) at a temperature range from *RT* to 450 °C and frequencies of measurement field (from 20 Hz to 100 kHz). DC conductivity measurements were registered in the temperature range from *RT* to 300 °C with a Keithley 6517B electrometer (Cleveland, OH, USA). Hysteresis (*P*–*E*) loops (at *RT*, for frequency 5 Hz) were examined with a Sawyer–Tower circuit and a Matsusada Inc. HEOPS-5B6 precision high-voltage amplifier (Kusatsu, Japan). The data were stored on a computer disc using an A/D, D/A transducer card (National Instrumental, Austin, TX, USA) and the LabView computer program. The magnetic properties were investigated using a SQUID (MPMS XL-7 Quantum Design, San Diego, CA, USA) magnetometer, within a temperature range from −271 °C to 30 °C, and a magnetic field of 0.1 T.

## 3. Results and Discussion

### 3.1. Crystal Structure and Microstructure Measurements

**Figure 1:**
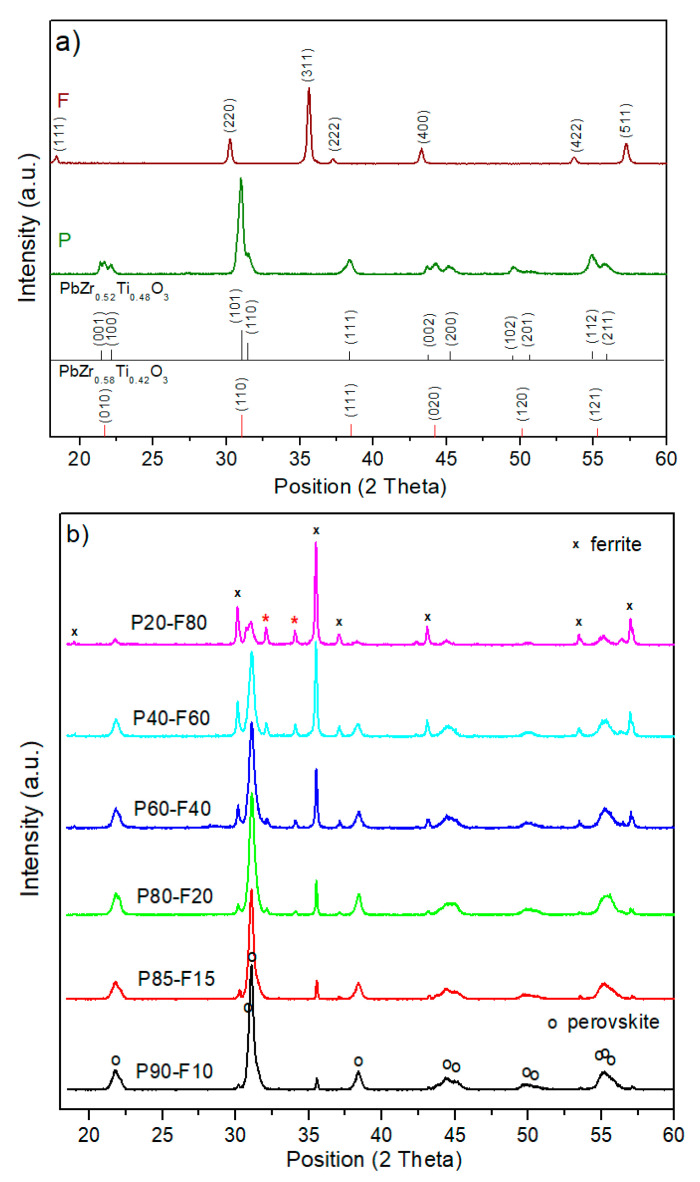
X-ray diffraction patterns of the (**a**) PZT-type material (P) and Ni_0.5_Zn_0.5_Fe_2_O_4_ materials (F), (**b**) P-F ceramic composites.

The X-ray investigations (Figure 1a) of the PZT-type ceramic powder (P) confirmed that at *RT*, the obtained materials have perovskite-type structure from morphotropic region (mix of tetragonal and rhombohedral phase). In the case of the tetragonal symmetry phase, the best match with experimental results to the PbZr_0.52_Ti_0.48_O_3_ material pattern (space group P4*mm*) [25] was obtained whereas in the case of the rhombohedral symmetry, the best match was obtained with the PbZr_0.58_Ti_0.42_O_3_ material pattern (space group R3*m*) [26]. In the case of the Ni_0.5_Zn_0.5_Fe_2_O_4_ ferrite material (F), the X-ray diffraction patterns at *RT* shows a cubic spinel phase. A good fit of the experimental results was obtained in the Ni_0.5_Zn_0.5_Fe_2_O_4_ material pattern (space group F*d*$\bar{3}$*m*) [27].

X-ray analysis of P-F ceramic composites (Figure 1b), with different percentages of their components, showed the presence of major maxima originating from both the ferroelectric phase (P material) and from the ferromagnetic one (F). With the increase in ferrite content in P-F composite samples, the intensity of reflections increases at the expense of the intensity of reflections derived from the perovskite phase. X-ray analysis also showed the presence of a small amount of secondary phase for group of samples with a greater amount of ferrite, i.e., P20-F80, P40-F60, P60-F40, and P80-F20 (marked with a red asterisk in Figure 1b). The good fit of the experimental data was obtained with the diffraction standard of the PbFe_12_O_29_ material with the hexagonal crystal system and space group P6_3_/*mmc*. This phase is formed during a high temperature sintering for the composite material with a lot of iron. In the case of the composite samples of P85-P15 and P90-P10, X-ray analysis showed no presence of secondary phases.

**Figure 2:**
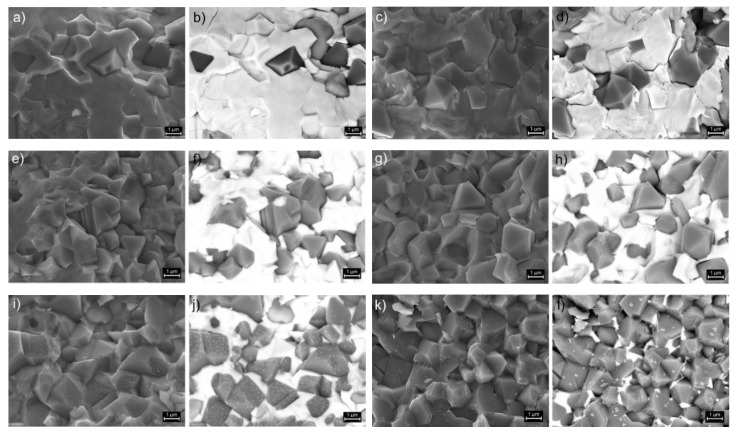
SEM images of the P-F ceramic composites taken in the standard SB mode (**a**,**c**,**e**,**g**,**i**,**k**) and BSE (**b**,**d**,**f**,**h**,**j**,**l**): (**a**,**b**) P90-F10, (**c**,**d**) P85-F15, (**e**,**f**) P80-F20, (**g**,**h**) P60-F40, (**i**,**j**) P40-F60, and (**k**,**l**) P20-F80.

Figure 2 shows microstructural SEM images of the P-F ceramic composite samples. SEM images were taken by two image capture techniques, i.e., standard SB technique and BSE technique (detection of backscattered electrons) and made for the same analysis area of the sample surfaces. The observation of BSE technique allows the visualization of differences in the microstructure of ferroelectromagnetic ceramic composite samples, i.e., the elements (phases) with a higher atomic number are shown as bright areas (ferroelectric component of the composite samples) while the elements (phases) with a lower atomic number are shown as dark areas (ferrite component of the composite samples). The use of the BSE technique in SEM studies of ferroelectromagnetic composites allows to visualize how the individual phases are distributed in the microstructure of the P-F ceramic samples and how the distribution of individual phases changes as a result of a change in their percentage in the composite. In the case of the P-F composite sample with the predominant ferroelectric perovskite phase (P90-F10), fine grains of the ferroelectric component surround the larger ferrite grains (Figure 2a,b). With the increase in the amount of ferrite in the P-F composite, an increase in the proportion of ferrite grains is observed in the microstructure, both in the form of single ferrite grains as well as the formed clusters of ferrite grains, locally creating a magnetic phase. In the case of the P-F composite sample with the dominant ferrite phase (P20-F80), numerous, fine ferrite grains surround the residual amount of the perovskite phase (Figure 2k,l).

The qualitative EDS analysis used to investigate the chemical composition of the P-F ceramic composite materials is presented in Figure S1 (Supplementary Materials). The microanalysis of the ceramic samples was performed on micro-areas of the fractures of samples (the results are the average value of 5 randomly selected sample areas). The qualitative EDS investigations confirmed the assumed share of the individual components, without any additional (foreign) elements. Depending on the increasing amount of ferrite in the P-F composite, an increase in the intensity of the peaks originating from its constituent elements (i.e., iron, zinc, and nickel) is observed in the EDS diagrams presented. At the same time, the intensity of peaks originating from the ferroelectric component phase (i.e., lead, zirconium, titanium, bismuth, niobium, and manganese) decreases. The EDS research has shown that P-F ceramic composites are free of impurities and that the chemical composition was relatively stable. The most stable chemical elements there are mainly elements of base ingredients, i.e., iron (Fe), nickel (Ni), zinc (Zn), lead (Pb), zirconium (Zr), and titanium (Ti). The experimental values differ slightly from the theoretical values; however, the chemical composition deviation is in an acceptable range for composite samples. Quantities of elements that are admixtures of the ferroelectric component of the P-F composites, i.e., P component, show the greatest discrepancies from the assumed theoretical values. In addition, the amount of some admixtures oscillates within the limits of the measurement error of a given element by the EDS probe.

**Figure 3:**
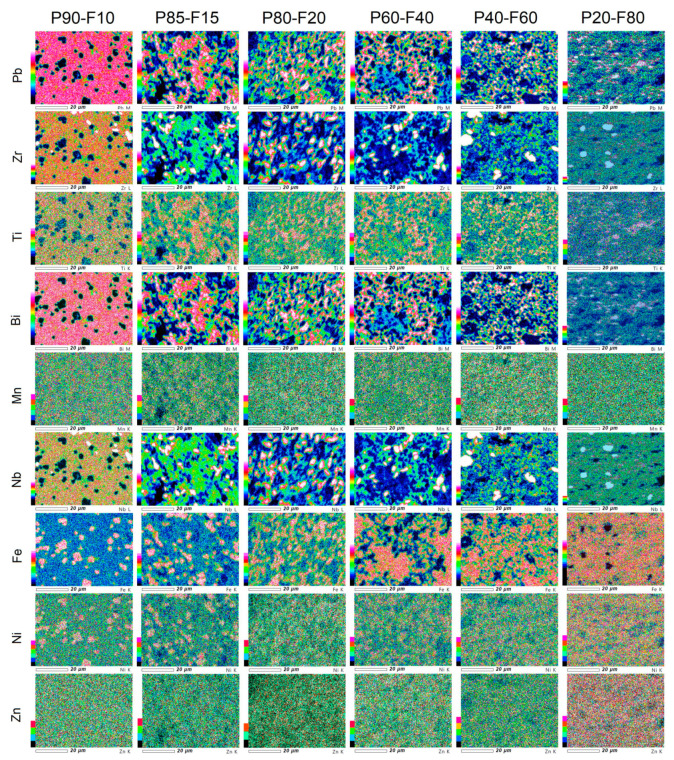
EPMA maps of the P-F ceramic composite.

The EPMA maps of the individual elements distribution of the P-F ceramic composites are depicted in Figure 3. EPMA analysis uses the X-ray emissions induced by an electron beam and was made on the surface of the samples. The analysis showed the distribution of individual elements in composite samples arranged according to the components of the composite phases, i.e., the ferroelectric and the magnetic phase. The EPMA analysis is consistent with the results of the EDS tests. In the areas of composite samples where these phases occur, the increased intensity of their individual components is visible. The most homogeneous distribution of the constituent elements is shown for the P90-F10 composite sample.

### 3.2. Magnetic Properties

**Figure 4:**
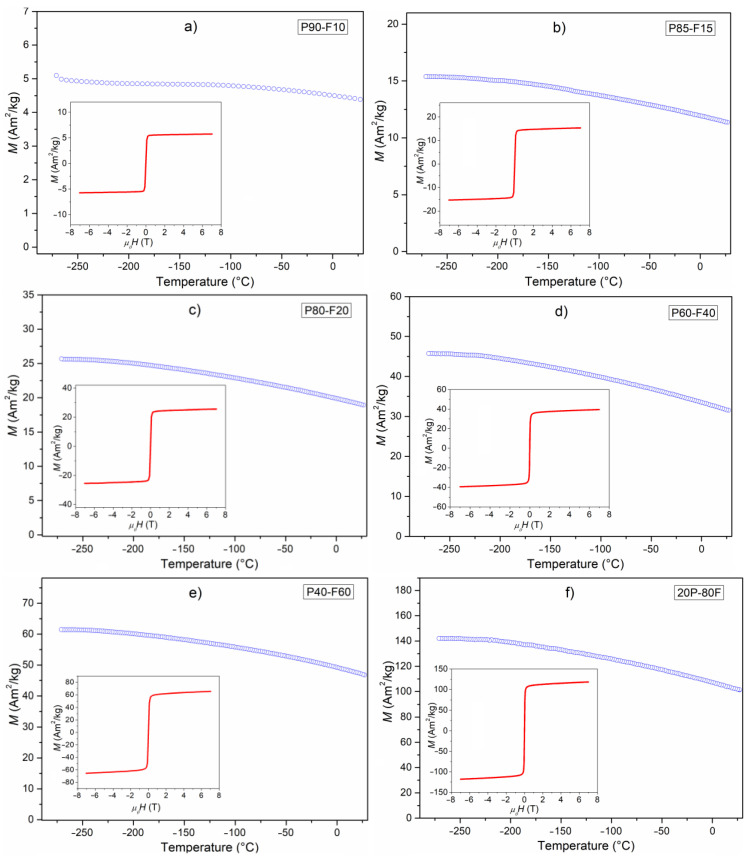
Temperature dependencies of magnetization for ceramic P-F composites: P90-F10 (**a**), P85-F15 (**b**), P80-F20 (**c**), P60-F40 (**d**), P40-F60 (**e**), and P20-F80 (**f**) (inset—magnetic hysteresis loops at *RT*).

Figure 4 shows the temperature dependencies of *M* magnetization in an external magnetic field of 0.1 T (in the temperature range from −270 to 30 °C) for a series of ceramic composite samples. For all composite samples, the highest values of magnetization occur at very low temperatures (−271 °C) and then begin to slightly decrease when the temperature rises. Temperature changes of magnetization show a dependence typical of ferroelectric–ferromagnetic composite materials, i.e., that consists of a dominating signal from the ferrimagnetic phase and a weak signal from the paramagnetic phase (a linear decrease of magnetization in a wide temperature range) [19].

**Figure 5:**
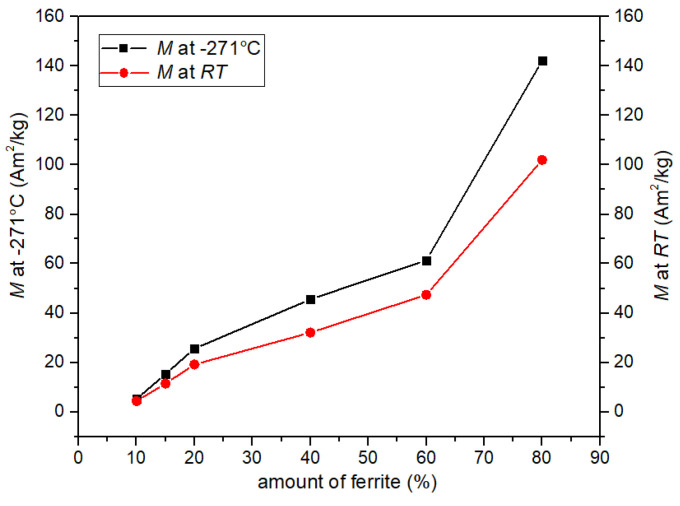
Influence of the amount of ferrite on the magnetization of the composite samples.

The highest values of magnetization at the temperature of −270 °C and at *RT* were revealed for the P20-F80 sample: 142 Am^2^/kg, and 102 Am^2^/kg, respectively. So, the decrease in the value of *M* is 71.7% of its initial value. Reducing the amount of ferrite in the composite significantly lowers the magnetization values (Figure 5 and Table 1). In the case of the sample P90-F10, the value of magnetization is very low, but the difference in the value of magnetization (at low temperature −271 °C and at *RT*) is not as large as in the case of the P20-F80 sample (there is a slow decrease in magnetization). The magnetization values at −270 °C and *RT* for sample P90-F10 are 5.2 Am^2^/kg and 4.4 Am^2^/kg, respectively (which is 84.6% of the initial value).

The appearance of the magnetic hysteresis loops for the P-F ceramic composite samples is shown in Figure 4 (inset). Very narrow magnetic hysteresis loops are observed at *RT*. The shape of the *M*(*H*) curves of P-F ceramic composite samples are typical for composite materials constructed based on soft ferrimagnetic ferrite [27]. Magnetic research has shown that the electric subsystem does not disturb the magnetic properties of the P-F composites, i.e., the magnetic properties increase adequately to the growth of ferrite in the composite samples.

### 3.3. Dielectric Properties

**Figure 6:**
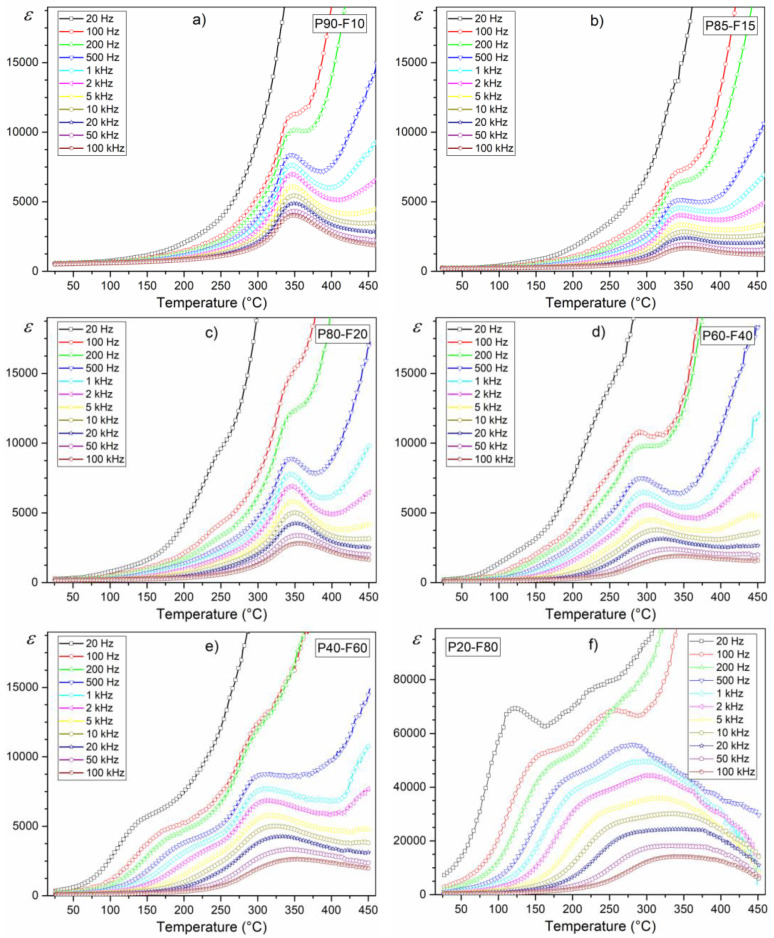
Temperature behavior of dielectric constant of the P-F ceramic composites: P90-F10 (**a**), P85-F15 (**b**), P80-F20 (**c**), P60-F40 (**d**), P40-F60 (**e**), and P20-F80 (**f**).

The multicomponent P ceramics have high electric permittivity values and the phase transition from ferroelectric to paraelectric phase occurs in a narrow temperature range [19]. The series of composite samples have high electric permittivity values (Table 1, Figure 6), but with much lower maximum electric permittivity values (at *T_m_*) compared to P ceramics. At *RT* (for frequency of 1 kHz) for the P90-F10 sample *ε_RT_* is 550, while for the P20-F80 sample, *ε_RT_* is 580. Temperature studies of the dielectric properties of a series of P-F composites revealed the occurrence of maxima originating from the ferroelectric–paraelectric phase transition (Figure 6), except that these maxima show a diffuse character (unlike for P ceramics).

**Figure 7:**
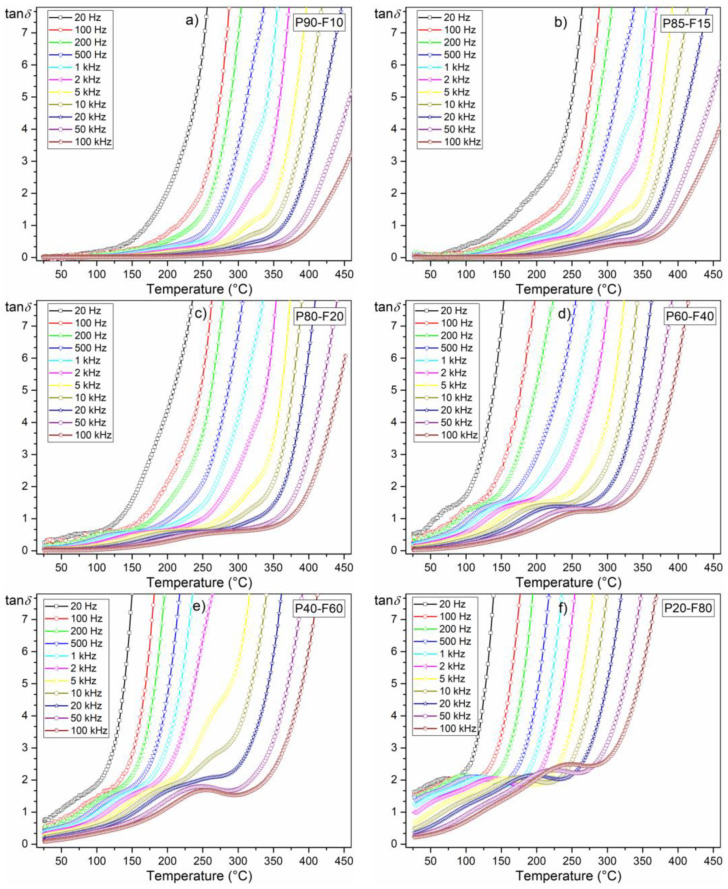
Temperature behavior of dielectric loss of the P-F ceramic composites: P90-F10 (**a**), P85-F15 (**b**), P80-F20 (**c**), P60-F40 (**d**), P40-F60 (**e**), and P20-F80 (**f**).

**Figure 8:**
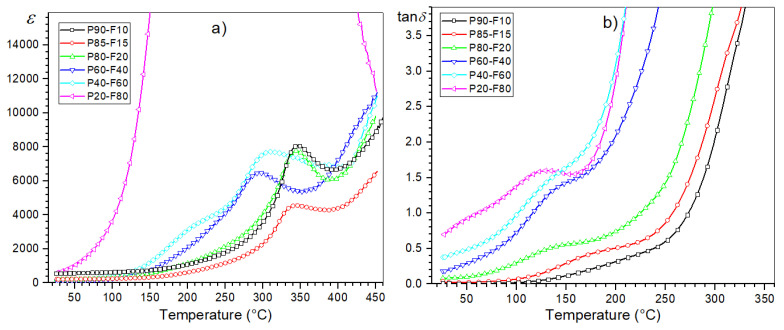
Summary temperature testing the dielectric properties of the P-F ceramic composites: electric permittivity (**a**), and dielectric loss (**b**).

For low ferrite content in the composite, this blurring is not large (P90-F10); however, as the percentage of ferrite in the composite increases further, this blurring broadens significantly. Also, the increase in ferrite reduces the maximum value of electric permittivity at *T_m_* and shifts this *T_m_* point towards lower temperatures (Figure 7 and Figure 8, Table 1). The completely extreme dielectric results are represented by the P20-F80 composite sample.

The degree of phase transition blurring in ferroelectric ceramic materials is affected by a number of factors depending on the technological process of their preparation. These include, inter alia, deviations from the composition in micro volumes of the composite, or inhomogeneity of the distribution of defects and mechanical stresses in the ceramic sample. These factors increase the differences in the phase transition temperatures of crystallites with different mechanical stress state and in crystallites with different chemical composition.

The greater the deviation from the chemical composition and the increase in the heterogeneity of the distribution of defects and mechanical stresses of crystallites, the greater the differences in the phase transition temperatures of individual crystallites. An additional factor affecting the blurring of ferroelectric phase transformation in ferroelectric composite compositions is the presence of a magnetic phase in the composite [19]. Ni_0.5_Zn_0.5_Fe_2_O_4_ ferrite at about 270 °C has a magnetic phase transition [28]. The influence of the magnetic sub-phase on the electrical sub-phase of ceramic composite materials is evident in the *ε*(*T*) diagrams in the form of disturbances of their normal waveforms at higher temperatures, and the greatest changes occur for the lowest frequency of the measuring fields.

The influence of the magnetic component (ferrite) in the composite is also visible on the tan*δ*(*T*) temperature plots of the composite samples, i.e., in the form of an increase in dielectric loss (Figure 7). At room temperature (for 1 kHz) for the P90-F10 composition, the tan*δ* value is 0.01, while for the P80-F20 composition, it is 0.68. For the compositions with high ferrite content, the dielectric loss in the whole measurement area is significantly high (Table 1, Figure 8b). In the summary graph, *ε*(*T*) and tan *δ*(*T*) (Figure 8a,b, respectively) for 1 kHz show the effect of the amount of ferrite on the dielectric properties in the ferroelectromagnetic composite samples. Increasing the ferrite in composite samples causes the electric permittivity to decrease, whereas the diffusion phase transition increases. Completely extreme high values of electric permittivity occur for P20-F80 composite sample. Additionally, increasing the ferrite in composite samples causes rapid growth of the dielectric loss, which is a negative phenomenon in terms of application possibilities.

Temperature tests of magnetic and dielectric properties for all P-F composite samples confirmed that at room temperature, the designed composite materials exhibit both magnetic (ferrimagnetic) and dielectric properties.

### 3.4. Ferroelectric Properties

**Figure 9:**
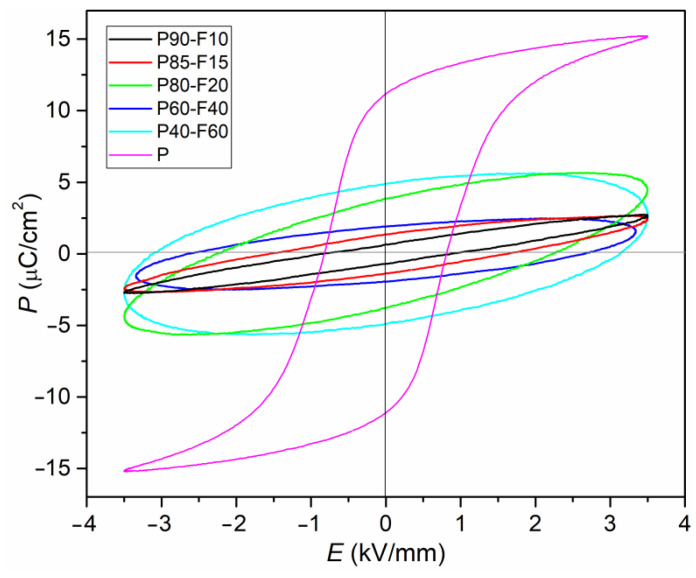
Hysteresis loops for the P-F ceramic composites (at *RT*, for 5 Hz).

Figure 9 shows electrical hysteresis loops for composite P-F samples, measured at *RT* and at a frequency of 5 Hz. For comparison, the figure also includes a hysteresis loop for P material that forms the basis ferroelectric component of the composite material. The *P–E* loop for P material demonstrate good saturation and is characteristic of ferroelectric soft materials, i.e., materials with majoritarian soft dopants. The presented plots of *P*(*E*) confirm that the magnetic addition (Ni_0.5_Zn_0.5_Fe_2_O_4_ ferrite) worsens the ferroelectric properties in the P-F composite. Figure 9 depicts that ferrite contributes to obtaining the shape of the electric hysteresis loop characteristic for linear dielectrics with losses. The loops are rounded and show no saturation for samples with a high ferrite content. With the increase in the ferrite in the composite composition, these effects intensify (and additionally, the leakage current also appears). The consequence of this is that for the P20-F80 sample, it was not possible to obtain a hysteresis loop at *RT* due to very high charge leakage currents.

### 3.5. DC Conductivity

**Figure 10:**
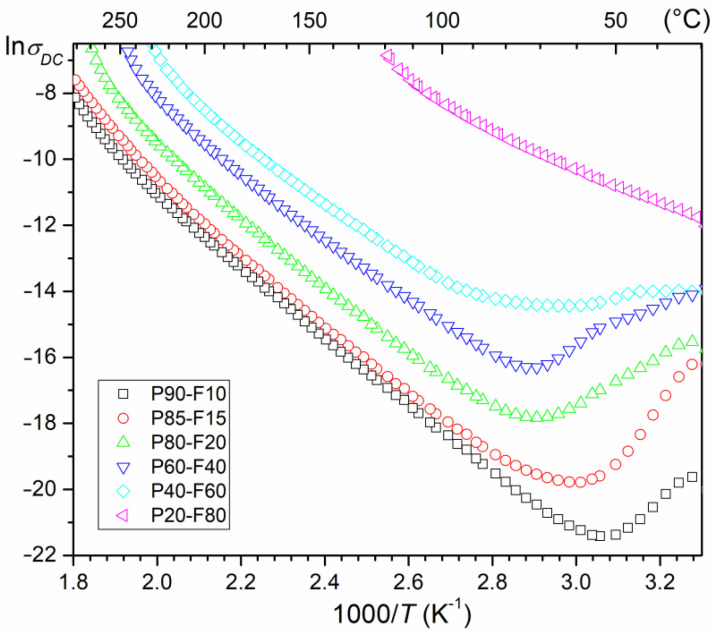
The ln*σ*(1000/*T*) relationship for the P-F ceramic composites.

At *RT*, the P-F composite samples have middle values of *ρ*_DC_ resistivity, i.e., from 9.59 × 10^6^ Ωm (for P90-F10) to 2.23 × 10^5^ Ωm (for P20-F80)—Table 1. The negative effect of the amount of ferrite in the P-F composite is also visible on the temperature ln*σ*_DC_(1000/*T*) curves of the composite samples in the form of an increase in electrical conductivity (Figure 10). The lowest DC electrical conductivity are exhibited by samples P90-F10 and P85-F15. As the amount of ferrite in the composite increases, the electrical conductivity definitely increases, and for the composition with the highest amount of ferrite (P20–F80), it is by far the highest over the entire temperature measurement region. Based on the slope of the curves ln*σ*_DC_(1000/*T*) and Arrhenius’ Equation (1), the activation energies *E_Act_* were calculated (in temperature regions with linear dependencies of the ln*σ*_DC_(1000/*T*) plots—Table 1).

(1) $$\sigma_{\mathrm{DC}}=\sigma_{0}\exp\left(\frac{E_{Act}}{k_{B}T}\right)$$

where *σ*_0_ is the pre-exponential factor, *k_B_* is the Boltzmann’s constant, *T* is the absolute temperature, and *E_Act_* is the activation energy. With the increase of the magnetic component in the P-F composite, a decrease in the activation energy value is observed (Table 1).

## 4. Conclusions

In this study, six ferroelectric-ferrimagnetic composite samples were prepared from a combination of PZT-type multicomponent material and nickel-zinc ferrite, with different percentages, i.e., 90/10, 85/15, 80/20, 60/40, 40/60, and 20/80. The synthesis of the composite compositions was performed by powder calcination route, while densification of the composite samples was carried out by the free sintering method (non-pressure sintering). In this study, two techniques were used to image the microstructure of P-F composite samples, i.e., SB and BSE technique, which allowed accurate visualization of the presence and distribution of the magnetic and ferroelectric component in the volume of the composites.

In the case of the P-F composite sample with the predominant ferroelectric phase (P90-F10), fine grains of the ferroelectric component surround the larger ferrite grains (F). With the increase in the amount of ferrite in the P-F composite, an increase in the proportion of ferrite is observed in the microstructure, both in the form of single ferrite grains as well as the formed clusters of ferrite grains. In the case of the composite sample of P-F with the dominant ferrite phase (P20-F80), numerous fine ferrite grains surround the residual amount of the ferroelectric phase (P).

The conducted tests have shown that at *RT*, ferroelectric–ferrimagnetic composites exhibit magnetic and electrical properties simultaneously. In the case of magnetic measurements, the electric subsystem does not disturb the magnetic properties of the P-F composites (the magnetic properties increase adequately to the growth of ferrite in the composite samples). Temperature changes of magnetization show a dependence typical of ferroelectric–ferromagnetic composite materials that consists of a dominating signal from the ferrimagnetic phase and a weak signal from the paramagnetic phase (a linear decrease of magnetization in a wide temperature range). The shape of the *M*(*H*) curves of P-F ceramic composite samples are typical for composite materials with soft ferrimagnetic ferrite. Temperature studies of dielectric properties, however, showed a negative effect of the magnetic subsystem on the electric permittivity and dielectric loss. There is also a significant increase in dielectric loss and electrical conductivity, and the electric hysteresis loop takes shape, characteristic of linear dielectrics with losses. For this reason, the composite samples with the highest content of ferroelectric component have the most favorable physical properties from the perspective of application possibilities. The occurrence of interdependence between the electrical and magnetic sub-systems of ceramic composites can be used, for example, to control the electrical properties—external magnetic field—and vice versa, control the electrical field with magnetic properties (e.g., used in new types of memory and electromagnetic converters).

The best set of physical properties of the multiferroics composite have ceramic samples with a dominant of ferroelectric component phase and a small amount of ferrite component (P90-F10). Such a multiferroic material retains the high ferroelectric properties of the ferroelectric component in the composite while also acquiring magnetic properties. 

## Figures and Tables

**Table 1:** Electrophysical properties of the P-F ceramic composites.

Parameter	P90-F10	P85-F15	P80-F20	P60-F40	P40-F60	P20-F80
*ρ* (g/cm^3^)	7.19	6.79	6.16	5.64	5.41	5.20
*ρ*_DC_ at *RT* (Ωm)	9.59 × 10^6^	4.57 × 10^6^	3.72 × 10^6^	5.77 × 10^5^	4.46 × 10^5^	2.23 × 10^5^
*T_m_* (°C) (1 kHz)	347	345	344	295	313	283
*ε* at *RT* (1 kHz)	550	220	173	108	129	580
*ε_m_* (1 kHz)	8080	4590	7750	6421	7767	42,342
tan*δ* at *RT* (1 kHz)	0.01	0.03	0.08	0.18	0.36	0.68
tan*δ* at *T_m_* (1 kHz)	6.35	6.46	9.86	10.80	25.38	31.90
*E_Act_* (eV)	0.96	0.93	0.89	0.82	0.77	0.49
*P_R_* (μC/cm^2^)	3.32	4.65	3.79	1.92	4.85	
*E_C_* (kV/mm)	1.51	2.02	2.23	2.60	3.11	
*M* at −271 °C (Am^2^/kg)	5.2	15.4	25.6	45.6	61.4	142.2
*M* at *RT* (Am^2^/kg)	4.4	11.5	19.2	32.1	47.4	102.0

## Data Availability

Data is contained within the article.

## Supplementary Materials

The following are available online at https://www.mdpi.com/article/10.3390/ma14102488/s1, Figure S1: EDS analysis of the P-F ceramic composites.
