# Supplementary figures and images for: Effect of Chemical Composition on Magnetic and Electrical Properties of Ferroelectromagnetic Ceramic Composites
Source: Materials (Basel). 2021 May 11;14(10):2488. doi: 10.3390/ma14102488 (PMC8151765; doi:10.3390/ma14102488)

# Supplementary Materials

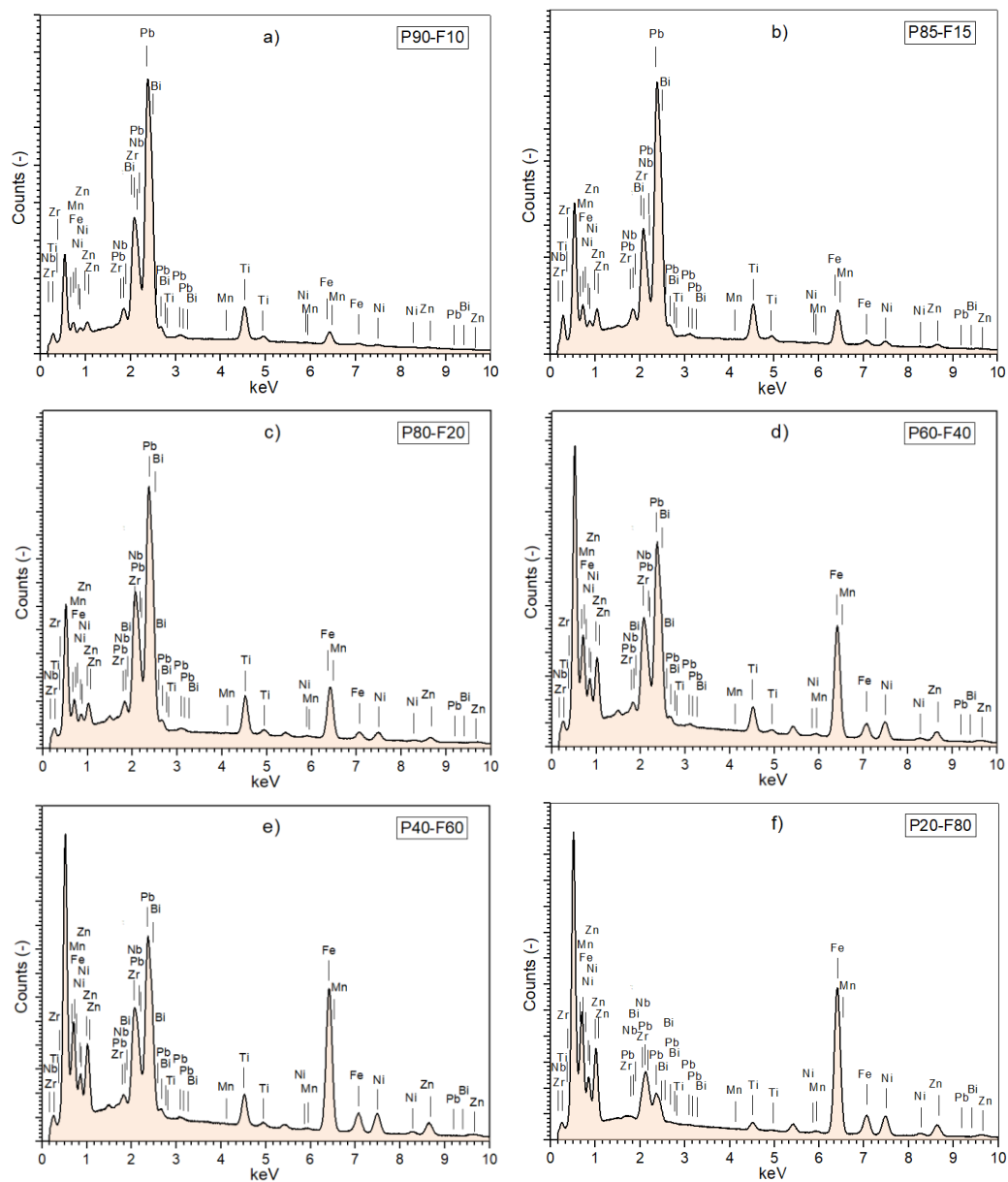

**Figure S1.** EDS analysis of the P-F ceramic composites.

Supplement: Supplementary file 1 [file materials-14-02488-s001.zip › materials-1208796-supplementary.pdf]
